# Production of High-Quality Red Fruit Juices by Athermal Membrane Processes

**DOI:** 10.3390/molecules27217435

**Published:** 2022-11-01

**Authors:** Rosanna Morelli, Carmela Conidi, Rosa Tundis, Monica R. Loizzo, Massimo D’Avella, Rosario Timpone, Alfredo Cassano

**Affiliations:** 1 Institute on Membrane Technology, ITM-CNR, 87036 Rende, Italy; 2 Department of Pharmacy, Health and Nutritional Sciences, University of Calabria, 87036 Rende, Italy; 3 Citrech Snc, Projects, Applications Consulting for Food and Citrus Industry, 98121 Messina, Italy

**Keywords:** red fruit juices, osmotic distillation, antioxidant activity, integrated membrane processes, athermal concentration

## Abstract

Membrane-based processes are increasingly used to clarify and concentrate thermo-sensitive fruit juices and plant extracts as alternatives to conventional processes. This work aimed to evaluate the quality of red fruit juices clarified and concentrated by an integrated membrane process with special regard to the preservation of valuable compounds. A red fruit juice obtained from a blend of pomegranate, cactus pear, and red orange juices of Sicilian origin was clarified by microfiltration (MF) and then pre-concentrated up to 33 °Brix by nanofiltration (NF). The pre-concentrated juice was finally concentrated by osmotic distillation (OD) up to 50 and 60 °Brix. Samples of clarified, pre-concentrated, and concentrated juice were analyzed for their physico-chemical composition and in terms of the antioxidant activity and inhibitory activity against α-amylase and lipase. The results clearly confirmed the assumption of a mild fruit juice processing method, allowing us to preserve the original nutritional and functional properties of the fresh juice. In particular, the OD retentate at 60 °Brix resulted the most active sample against pancreatic lipase and α-amylase inhibitory activity with IC_50_ values of 44.36 and 214.65 μg/mL, respectively.

## 1. Introduction

Fruit juices are nutritional beverages that play a significant part in a healthy diet since they are enriched in nutrients covering a wide range of minerals, proteins, and protective antioxidants such as vitamins, phenolic, and carotenoid compounds. Their global market size is 48.6 billion liters, and the market is projected to expand at a CAGR of 2.1% for the forecast period 2021–2026 [1].

Dietary intake of fresh fruits and vegetables have clear effects against a number of chronic diseases such as obesity, cardiovascular, and neurodegenerative diseases, cancer, inflammatory disorders, diabetes, viral infection, stroke, Alzheimer’s, and oxidative stress-induced other malignancies [2]. In addition, the prevailing pandemic of COVID-19 has dramatically elevated consumer demand for fruit juices as immunity-boosting foods [3].

At present, there is a great interest in studying the contribution of diet to the prevention and/or treatment of type 2 diabetes, obesity, and metabolic disorders associated with metabolic syndrome (MetS), a condition characterized by several risks including atherogenic dyslipidemia, hyperglycemia, abdominal obesity, and high blood pressure [4].

Diabetes mellitus is a global health problem. In 2019, 463 million people suffered from diabetes worldwide. Its global prevalence is increasing. The inhibition of carbohydrate digestive enzymes is one of the strategies to reduce the post-prandial hyperglycemia in patients affected by type 2 diabetes [5]. Some inhibitors of these enzymes, acarbose, miglitol, and voglibose, have been approved for medicinal use and some classes of natural compounds, in particular, flavonoids have been demonstrated to exhibit a similar inhibitory activity [6].

Pancreatic lipase is the most important enzyme in the digestion of triglycerides. The reduction in energy intake from dietary fat through the inhibition of this enzyme may be a therapeutic approach for the treatment of obesity. Several polyphenols have shown interesting inhibitory effects on this enzyme [7,8,9].

Thermal processes such as pasteurization, sterilization, and concentration by thermal evaporation under vacuum are traditionally used in the fruit juice processing industry. Unfortunately, these processes lead to a remarkable change in the juice quality due to the degradation of nutritional and sensorial components and the appearance of some “cooked” notes recognized as off-flavors [10,11]. Several non-thermal technologies have emerged in the past years as alternatives to traditional thermal techniques in order to meet the current demand of minimally processed fruit juices with considerable shelf stability [12]. They include ultrasound, high pressure, pulsed light, pulsed electric field, and ozone processing [3] as well as membrane technology [13]. Among these techniques, membrane filtration is an alternative method that reduces the heat-associated loss of nutritional and functional quality due to the moderate operating temperatures used in the process. Additional advantages include high selectivity based on unique separation mechanisms, no use of chemical additives, easy scale-up, compact and modular design, and low energy consumption [14]. In particular, pressure-driven membrane operations including microfiltration (MF), ultrafiltration (UF), nanofiltration (NF), and reverse osmosis (RO) represent well-established technologies for the clarification, fractionation, demineralization, and concentration of additive-free high-quality fruit juices with a natural fresh taste [15]. Other membrane processes such as membrane distillation (MD) and osmotic distillation (OD), classified as membrane-contactor techniques, represent attractive processes for the concentration of solutions containing thermosensitive compounds such as fruit juices and pharmaceuticals, since they operate under ambient temperature and pressure, thereby maintaining original organoleptic and sensorial characteristics of the product. Unlike pressure-driven membrane operations, MD and OD are driven by a vapor pressure difference between porous hydrophobic membrane surfaces, through which only water vapor molecules can pass. These processes are characterized by several advantages over conventional membrane processes including low fouling index, a high retention of solutes, and the possibility to treat solutions with high viscosity. In addition, since the driving force is not a hydraulic pressure difference, these processes allow us to reach high solute concentrations, similar to those obtained in thermal evaporation [16].

Finally, technological innovations in fruit juice processing can be achieved through the implementation of hybrid or integrated membrane systems that combine several membrane separation steps in a multistage configuration. This combination leads to technological and economic benefits that cannot be achieved when developed as one concept, offering new and interesting opportunities for the production of innovative functional fruit and vegetable juices [17].

In this context, this work aimed at investigating, for the first time, a non-thermal concentration of a blend of cactus pear, pomegranate, and blood orange juices for the production of a functional juice with improved antioxidant activity that is potentially able to counteract type 2 diabetes and obesity. The red juice was clarified by microfiltration (MF) and then pre-concentrated by nanofiltration (NF) up to 33 °Brix. Concentrated juices at 50 °Brix and 60 °Brix were obtained from the pre-concentrated juice by using osmotic distillation. Samples of clarified, pre-concentrated, and concentrated juices were analyzed for their physico-chemical parameters as well as for their inhibitory activity against α-amylase and lipase and antioxidant effects.

## 2. Results and Discussion

### 2.1. Performance of OD Membrane

**Figure 1:**
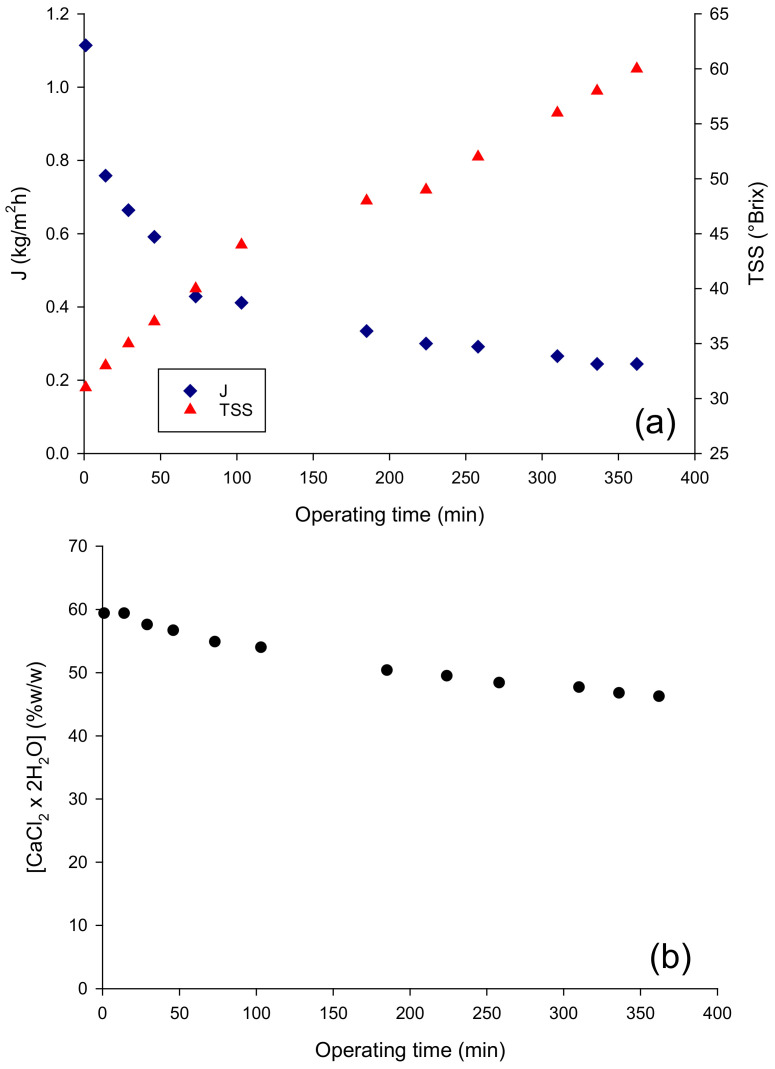
Concentration of pre-concentrated red fruit juice by OD. Time evolution of (**a**) the evaporation flux and (**b**) brine concentration.

The performance of the OD membrane in terms of evaporation flux in the concentration of the pre-concentrated juice up to 60 °Brix is depicted in Figure 1a: the initial evaporation flux, of about 1.11 kg/m^2^ h, decreased gradually to reach a final value of 0.24 kg/m^2^ h. Evaporation fluxes of the same order by using a Liqui-Cell Extra-Flow membrane contactor have also been reported in the concentration of clarified pomegranate juice [18], cranberry juice [19], roselle extract, apple, orange and grape juices [20,21]. The decrease in evaporation flux observed in the OD process can be attributed only in part to the dilution of the brine solution, which in turn leads to the decrease in the vapor pressure of the osmotic agent and, consequently, to a decrease in the driving force for water transport from the feed through the membrane. Indeed, the observed reduction in the evaporation flux, of approximately 78%, corresponded to a much lower decrease in the brine concentration (about 22%) (Figure 1b). This result can be attributed to an additional effect of the juice viscosity, which increased exponentially with the juice concentration, leading to an increase in the resistance to mass transfer in the liquid phase and, consequently to the polarization effects that reduce the driving force of the process.

### 2.2. Physico-Chemical Parameters of Processed Juice

The analyses of the total soluble solids (TSS), suspended solids (SS), total color density (TCD), pH, and conductivity in samples of red fruit juices processed by MF, NF, and OD are reported in Table 1. The clarified juice presented a concentration of soluble solids of 13 °Brix, slightly lower than that of the juice. This result is attributable to the presence of suspended solids in the juice, which interferes with the measurement of the refractive index [22]. The MF process allowed for a complete removal of the suspended solids from the raw juice with the production of a clear and limpid juice. Regarding the color analysis, there were no particular differences between the concentrated juice and both samples of the raw (feed MF) and clarified juice (permeate MF). Therefore, it can be assumed that in the process of clarification, pre-concentration, and concentration, the color of the starting juice is totally preserved due to the low temperatures achieved during membrane-based treatments. On the other hand, a decrease in red color intensity was observed by Onsekizoglu [23] in thermally evaporated pomegranate juice attributed to the degradation or polymerization of anthocyanins at high temperatures. As for the color, the pH of the samples coming from the different steps of the process remained practically unchanged.

A slight increase in the electrical conductivity was observed in the clarified juice in comparison to the raw juice. However, in the subsequent steps of the process, the conductivity of the retentates at 33, 50, and 60 °Brix was practically similar. This result, in relation to the concentration process by OD, clearly indicates that salt compounds are not transferred from the brine solution (calcium chloride dihydrate) to the juice and that both hydrophobicity and integrity of fibers during the process is well-preserved.

The analysis of betalains (betacyanins and betaxhantins) in juice samples from the MF-NF-OD sequence is shown in Table 2. The pre-concentration process of the clarified juice produced a significant increase in betaxanthins compared to betacyanins. Concentrated samples at 60 °Brix showed a slight increase in both betacyanins and betaxanthins compared to concentrated samples at 50 °Brix and with respect to the clarified juice. Similar to data reported by Cassano et al. [24] in the OD process of yellow variety of cactus pear juice, the betacyanin/betaxanthin ratio remained constant, regardless of the degree of concentration obtained.

The results related to the determination of polyphenols, flavonoids, and anthocyanins in the samples from the MF, NF, and OD processes are shown in Table 3. By referring to the analysis of polyphenols, the starting juice showed a value of 1370.2 mg GAE/L. This value was higher than that found in the blood orange juice of a Tarocco variety of Calabrian origin (907.2 mg GAE/L) [25] and lower than that measured for pomegranate juice of Sicilian origin (*Punica granatum* L. cv Selinunte) (about 1500 mg catechin/L) [26]. Although there was a slight decrease in polyphenols (about 2%) in the clarified juice, the concentrated juice at 60 °Brix had a polyphenol content comparable to that of the starting juice.

By referring to the flavonoid content, the analysis of the data showed a 2% reduction in these components in the juice clarified by MF compared to the starting juice; a further loss of 4% was observed in the juice pre-concentrated by NF. On the other hand, the OD process did not substantially modify the flavonoid content regardless of the degree of concentration reached. In fact, in both samples at 50 °Brix and 60 °Brix, the flavonoid content was practically similar to that of the pre-concentrated juice.

The MF process determined a reduction in anthocyanins in the starting juice of 12% (from 408 to about 362 ppm). A further reduction of the same amount was observed in the subsequent pre-concentration step. The retentates of the OD process, at 50 and 60 °Brix, had the same anthocyanin content as the pre-concentrated juice. Reductions in the anthocyanin content of about 10% have been also reported by Galaverna et al. [27] in the pre-concentration of blood orange juice by reverse osmosis.

All of these data clearly indicate that the OD process did not alter the polyphenol and anthocyanin content of the processed juice. Similar results were obtained in the OD concentration of blood orange [27,28], pomegranate [18], and bergamot [29] juices from clarified juices by UF. On the other hand, data reported in the literature indicate a strong degradation of anthocyanin pigments during the concentration of pomegranate juice by thermal treatment, accompanied by the presence of significant levels of 5-hydroxymethyl furfural (indicator of potential browning of the juice) and the reduction of mineral salts such as sodium, iron, lead, copper, and zinc between 44% and 69% [30]. Furthermore, heating/evaporation processes (microwave heating, rotary vacuum evaporator, and evaporation at atmospheric pressure) used for the production of concentrated pomegranate and apple juices significantly modified the color parameters (*L*, *a*, and *b*) of the final product [31,32].

### 2.3. Enzymes Inhibitory Activities

We investigated the ability of red juices to inhibit pancreatic lipase, one of the most widely studied targets for extracts and/or pure compounds as anti-obesity agents because it is responsible for the hydrolysis in the intestinal lumen of 50–70% of total dietary fats and α-amylase, whose inhibitors are considered as a viable prophylactic treatment of hyperglycemia [33,34].

All red juices inhibited both enzymes in a concentration-dependent manner. The IC_50_ and half-inhibitory concentration values are reported in Table 4.

Interesting IC_50_ values in the range of 44.36–48.82 μg/mL (in comparison to the positive control lipase with an IC_50_ value of 37.15 μg/mL) were found against lipase. The retentate at 60 °Brix (retentate OD (2)) was the most active, followed by the retentate at 50 °Brix (retentate OD (1)).

The investigated juices were less active as α-amylase inhibitors with IC_50_ values ranging from 214.65 μg/mL for retentate OD (2) and 304.06 μg/mL for feed MF. Additionally against α-amylase, the most active sample was retentate OD (2) (IC_50_ of 214.65 μg/mL), followed by retentate OD (1) (IC_50_ of 252.62 μg/mL) and the retentate of the NF process (IC_50_ of 258.16 μg/mL).

Analyzing all of the obtained data, we can conclude that, even without evident differences, the processed juices showed a better inhibitory activity of both the amylase and lipase enzymes. In a recent study, Berger et al. [35] analyzed the inhibitory activity against α-amylase and α-glucosidase of various extracts prepared from juices, concentrates, and purees of nine different berries, which differed in their anthocyanin and co-pigment profile. It was found that extracts with the highest phenolic content also showed one of the highest inhibitory activities.

### 2.4. Antioxidant Activity

**Figure 2:**
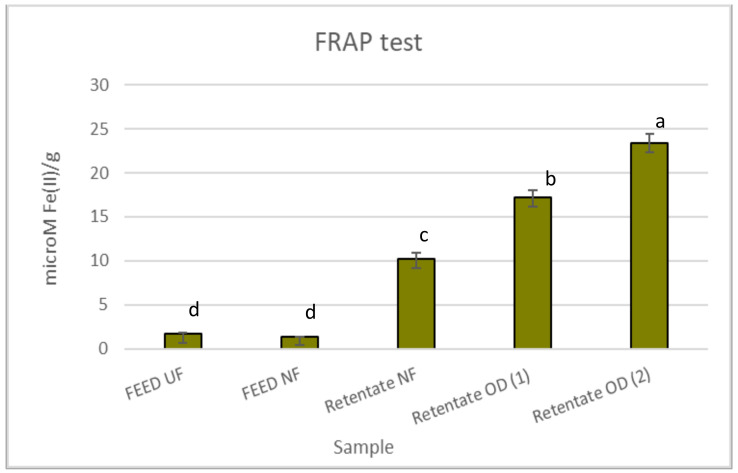
Antioxidant activity of red juices in the FRAP test. Data are reported as the mean ± standard deviation (*n* = 3). Butylhydroxytoluene (BHT) was used as the positive control (63.20 ± 1.94 µM Fe(II)/g). Differences between samples were evaluated by one-way analysis of variance (ANOVA) test completed with a multi-comparison Tukey’s test (*p* < 0.01). Means with different small letters differ significantly.

Figure 2 reports the antioxidant effects of red juices assessed by the FRAP test. The retentate fractions were the most active with values of 10.16, 17.18, and 23.33 µM Fe(II)/g for the NF retentate, retentate OD (1), and retentate OD (2), respectively. However, generally, all juices were less active than the positive control BHT (63.20 µM Fe(II)/g).

**Figure 3:**
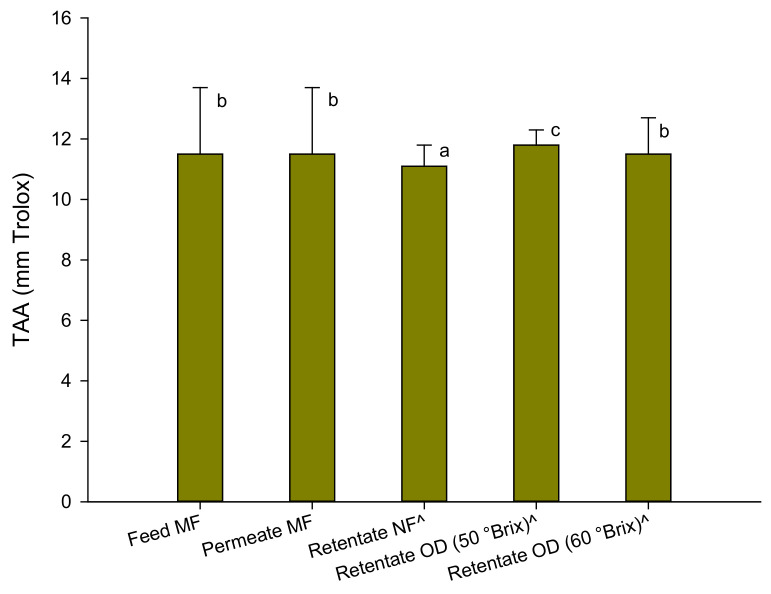
Antioxidant activity of red juices in the ABTS test. Data are reported as the mean ± standard deviation (*n* = 3). ^ Samples diluted at 13 °Brix. Differences between samples were evaluated by one-way analysis of variance (ANOVA) test completed with a multi-comparison Tukey’s test (*p* < 0.01). Means with different small letters differ significantly.

The results of the antioxidant activity assessed by the ABTS test are depicted in Figure 3.

The antioxidant activity of the raw juice was comparable to that found in the pomegranate juice of Sicilian origin (*Punica granatum* L. cv Selinunte) [18] and from pomegranate arils of the Wonderful variety grown in California (12–14 TEAC) [36]. Slightly lower values (of the order of 8.65 mm Trolox) were measured in the Tarocco variety blood orange juices grown in Sicily [27].

The clarification process did not produce any changes in the total antioxidant activity of the juice. A slight decrease (about 4%) of the total antioxidant activity was found in the pre-concentrated juice at 33 °Brix, while the OD process did not substantially determine a loss of antioxidant components compared to the pre-concentrated juice, also at a higher concentration degree. These results are in agreement with the results of the polyphenol content which, as well-known, contribute to a large extent to the antioxidant activity of the juice. In addition, the results obtained in the concentration of other fruit juices such as pomegranate [18], kiwi [37], blood orange [27], and cactus pear [24] juices with the same OD membrane module used in the present study confirm the efficiency of the process in preserving the antioxidant activity of the juice in comparison to conventional thermal processes.

## 3. Materials and Methods

### 3.1. Red Fruit Juice

The red fruit juice used for the experimental activities was produced on a semi-industrial scale as a blend of blood orange, prickly pear, and pomegranate juice by Citrofood Srl (Capo d’Orlando, Messina, Italy). The orange juice was sampled in the period of full ripeness in a company equipped with an FMC in-line extractor; this is an extraction system working on the single fruit, obtaining the juice from the squeezing of the endocarp only and minimizing the amount of the essential oil dissolved in solution. The pomegranate juice used in the mixture was obtained by directly sending pomegranate arils—the juicy seeds separated from the skins by means of mechanical separation—on a centrifugal helical screw separator equipped with hole separators of different sizes. For prickly juice, an extraction machine of citrus origin was specially modified in order to remove the peel from the fruit before pressing. A high-quality prickly pear juice was obtained once the raw product was refined and the seeds removed.

The juice blend was prepared from all the not from concentrate (NFC) juices mixed according to the following weight ratios: 65% blood orange juice, 25% pomegranate juice, 10% prickly pear juice. These weight ratios were fixed on the basis of organoleptic considerations. In particular, the pomegranate juice used was of the Wonderful variety, which is very rich in anthocyanins but also in tannins and its dosage was balanced according to the final taste of the blend. On the other hand, the low dosage of the prickly pear juice was established in relation to its lower decisive taste in comparison with orange and pomegranate juices. The components, still frozen, were milled with a special crusher, sent to a tank to ensure homogeneity, and pre-refined by means of a blade refiner. The produced juice (about 900 kg) was stored in an intermediate bulk container (IBC) and submitted to the clarification step.

### 3.2. Juice Clarification and Pre-Concentration

The clarification step was performed by using a microfiltration (MF) pilot plant consisting of feed and washing tanks, feed and recirculation pumps, and a tubular heat exchanger. The plant was equipped with two multichannel ceramic membranes (Model type 19/6) supplied by Atech Innovations GmbH (Gladbeck, Germany), each one with a length of 1200 mm, a pore size of 0.2 μm, and a specific filtration area of 0.42 m^2^. The MF system was operated at a transmembrane pressure (TMP) of 4.5 bar and a temperature of 20 ± 5 °C. The average permeate fluxes were about 140 L/m^2^ h.

The clarified juice was pre-concentrated by nanofiltration (NF) by using a NF plant equipped with two spiral-wound membrane modules (TFC 8040-SR-375) supplied by Kock Glitsch Italia Srl (Vimercate, Italy) with a molecular weight cut-off (MWCO) of 200 Da and a total membrane surface area of 69.6 m^2^. The NF system was operated at a TMP of 34 ± 2 bar, a temperature of 20 ± 5 °C, and a feed flowrate of 7.8 ± 0.1 m^3^/h up to a volume concentration factor of 2.5. Permeate fluxes obtained in the selected operating conditions were in the range of 0.7–2.2 L/m^2^ h. The obtained pre-concentrated juice (about 310 kg) was transferred in aseptic bags of 20 L and stored at −18 °C before the final concentration. Both the MF and NF systems were operated according to the batch concentration configuration in which the permeate stream was collected separately while the retentate was continuously recycled back to the feed tank.

### 3.3. Juice Concentration

Osmotic distillation was carried out in batch mode in a lab scale plant supplied by ITEST s.r.l. (Corato, Bari, Italy) consisting of two independent circuits, one for the juice and the other for the brine. The plant was equipped with a Liqui-Cell Extra-Flow 2.5 × 8-in. membrane contactor (Membrana, Charlotte, NC, USA) containing microporous polypropylene hollow fibers with an average pore diameter of 0.2 μm and a total membrane surface area of 1.4 m^2^. The pre-concentrated juice was pumped through the shell side of the membrane module, while in the fiber lumens (tube side) flowed a 60 *w*/*w*% calcium chloride dihydrate (Fluka Chemie GmbH, Buchs, Switzerland) solution using two independent magnetic drive gear pumps with variable motor velocity. Feed and stripping solution were recirculated through the contactor in a counter-current mode at an average flow rate of 1.9 ± 0.1 L/min and 1.0 ± 0.1 L/min, respectively. The OD system was operated at a temperature of 25 ± 2 °C whereas the average pressure in both compartments was about 0.6 ± 0.1 bar.

Both solutions were recirculated back to their reservoirs after passing through the contactor, at a temperature of 25 ± 2 °C.

Inlet and outlet pressures for both the tube side and shell side streams were registered by pressure gauges in order to control the pressure differentials between the two sides of the membrane. For each experimental run, 8 kg of brine and 6–10 kg of pre-concentrated juice were used. Each run was stopped when the concentration of soluble solids was 50 or 60 °Brix. The flowrate of extracted water during the concentration process was calculated by measuring the weight loss of the juice over time by a balance (Gibertini Elettronica, Milan, Italy) placed under the juice tank. Flow rates normalized by the surface area of the membrane provided the evaporation flux (J_w_) values.

**Figure 4:**
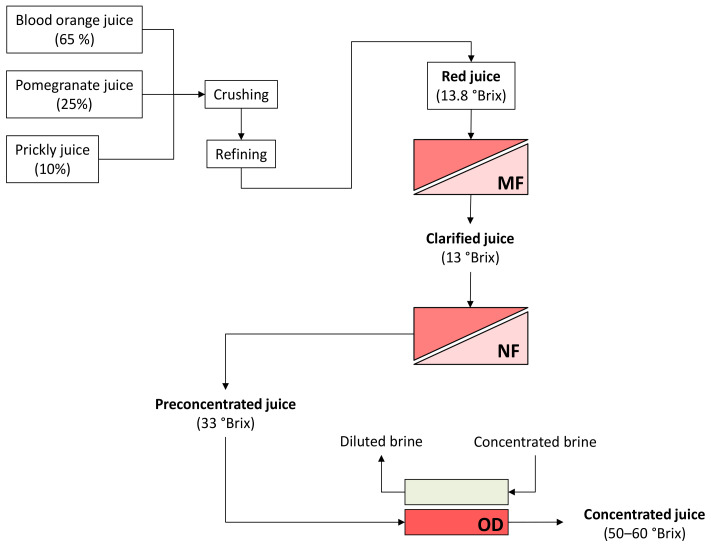
Schematic layout of the investigated process. MF, microfiltration; NF, nanofiltration; OD, osmotic distillation.

A schematic layout of the investigated process is depicted in Figure 4.

### 3.4. Physico-Chemical Analysis

The feed, permeate, and retentate samples were analyzed for their content of total soluble solids, pH, total color density, total phenolic compounds, total flavonoids, anthocyanins, and betalains. To facilitate the comparison of data between different samples, the NF retentate sample (at 33 °Brix) and the OD retentate samples (at 50 and 60 °Brix) were diluted to the same soluble solid concentration of the clarified juice (MF permeate at 13 °Brix).

#### 3.4.1. Total Soluble Solids

Total soluble solids (TSS) were carried out using hand refractometers (Atago Co., Ltd., Tokyo, Japan) with a scale range of 0–32, 28–62, and 58–90 °Brix.

#### 3.4.2. pH, Electrical Conductivity and Suspended Solids

pH was measured by an Orion Expandable ion analyzer EA 920 pH meter (Allometrics, Inc., Baton Rouge, LA, USA) with automatic temperature compensation. The electrical conductivity was measured by a Five Easy FE30 conductivity meter (Mettler-Toledo S.p.A., Milano, Italia). The suspended solid content was determined in relation to the total juice (%, *w*/*w*) by centrifuging, at 2000 rpm for 20 min, 45 mL of a pre-weighted sample; the weight of settled solids was determined after removing the supernatant.

#### 3.4.3. Total Color Density

Total color density (TCD) was expressed as the total values of absorbance of the brown compounds, which showed maximum absorbance at 420 nm and the absorbance of fruit juice, which reached the maximum at 533 nm [38]. The clarified juice samples were diluted with distilled water to achieve an absorbance of less than 1.0 at 533 nm. The TCD was estimated according to the following equation:

(1) $$\mathrm{TCD}=\left[\left(A_{420}+A_{533}\right)-2\left(A_{700}\right)\right]*F_{D}$$

where the absorbances at 420, 533, and 700 nm are those relating to the wavelength measured for each sample, while *F_D_* is the dilution factor.

#### 3.4.4. Total Phenolic Compounds

Total phenolic compounds (TPC) were estimated colorimetrically by using the Folin–Ciocalteau method [39], which is based on reducing tungstate and/or molybdate in the Folin–Ciocalteau reagent by phenols in an alkaline medium, resulting in a blue-colored solution. Results were expressed as mg gallic acid equivalents per g of sample dry weight (mg GAE/g).

#### 3.4.5. Total Flavonoids

The flavonoid content was determined according to the colorimetric Davis method [40]: 7 mL of diethylene glycol were added to 0.5 mL of sample and 0.5 mL of approximately 4 N sodium hydroxide; the increase in color was read at 420 nm after 20 min. The observed color increases were compared with a standard curve prepared from pure hesperidin. The results were expressed as mg hesperidin L^−1^.

#### 3.4.6. Anthocyanins

Anthocyanins were determined by a colorimetric method [41]: 5 mL samples of press liquor were mixed with 45 mL of a EtOH/HCl solution prepared by mixing 79.3 mL of anhydrous ethyl alcohol with 20.3 mL of HCl (37%). The absorbance was measured at 535 nm. The calibration curve was obtained by measuring the absorbance of standard solutions of pure cyanidin-3 glucoside. The anthocyanin concentrations were calculated using the extinction coefficient 1018.3 at 535 nm.

#### 3.4.7. Betalains

The quantification of betalains was carried out in deionized water without pH adaptation by applying the molar extinction coefficients of betanin (e = 60,000 L/mol cm in H_2_O; k = 538 nm; *MW* = 550 g/mol) and indicaxanthin (*ε* = 48,000 L/mol cm in H_2_O; λ = 480 nm; *MW* = 308 g/mol). The juice was diluted with deionized water to obtain absorption values of 0.8 ≤ *A* ≤ 1.0. The betalain content (*BC*), expressed as mg/L, was calculated by using the following equation:

(2) $$BC=\frac{A\cdot DF\cdot MW\cdot 1000}{\varepsilon \cdot L}$$

where *A* is the absorption at 538 and 480 nm for betacyanins and betaxanthins, respectively; *DF* is the dilution factor; and *L* is the pathlength of the 1-cm cuvette. For *MW* and *ε*, the molecular weights and extinction coefficients of the representative compounds betanin and indicaxanthin have to be considered, respectively [42].

### 3.5. Enzymes Inhibitory Activities

#### 3.5.1. Pancreatic Lipase Inhibitory Activity

The pancreatic lipase inhibitory activity of red juices was determined as previously reported [43]. In brief, a 5 mM solution of 4-nitrophenyl octanoate (NPC) in dimethyl sulfoxide (DMSO), an aqueous solution of lipase (1 mg/mL), and Tris-HCl buffer (pH 8.5) were prepared. The mixture consisting of enzyme, NPC, red juices at different concentrations, and buffer was incubated for 30 min at 37 °C. Then, the absorbance was read at 405 nm. Orlistat was used as a positive control.

#### 3.5.2. α-Amylase Inhibitory Activity

The α-amylase inhibitory test was conducted following the previously reported procedure [44]. Briefly, the α-amylase solution was prepared by dissolving the enzyme (25.3 mg) in cold distilled water (100 mL). The addition of a 3,5-dinitrosalicylic acid solution to a sodium potassium tartrate solution allowed us to prepare the colorimetric reagent. Red juices (concentrations in the range 12.50–1000 mg/mL) were added to the starch solution and left to react with the enzyme at room temperature. The absorbance was measured at 540 nm and acarbose was used as a positive control.

### 3.6. In Vitro Antioxidant Activity

Assessing the antioxidant activity is essential in ensuring the quality of foods, but also in studying the efficiency of antioxidants in preventing and treating diseases related to oxidative stress such as diabetes, cancer, and neurodegenerative diseases [45].

A number of assays have been developed to measure the radical scavenging capacity, reducing power, and other specific attributes of antioxidants at the molecular or cellular levels as well as overall oxidation inhibition in more complex food and biological systems. These methods vary in terms of antioxidant mechanism, oxidation initiator, substrate type, result expression, and ease of operation.

The appropriate selection of the method or the combination of methods is a key step for the effective assessment of antioxidant activity, and ultimately, the potential of the antioxidants as health promoting agents or food preservatives [46].

Herein, we investigated our samples by using the following tests: ferric reducing activity power (FRAP) and the ABTS/potassium persulfate decolorization assay.

#### 3.6.1. FRAP Test

The ferric reducing activity power (FRAP) test is a method to measure the reduction in the complex of ferric ions (Fe^3+^)-ligand to the intensely blue ferrous complex (Fe^2+^) by means of antioxidant agents in acidic environments.

The test uses tripyridyltriazine (TPTZ) as the linking ligand to the iron ion [47]. After 30 min of incubation at room temperature, the absorbance was read at 595 nm. Antioxidant activity was determined as an increase in absorbance, and the results were expressed as micromolar equivalents of Fe^2+^. Samples were tested at the concentration of 2.5 mg/mL. The positive control was butylated hydroxytoluene (BHT).

#### 3.6.2. ABTS Test

In the ABTS test, the radical monocation of the 2,2′-azino-bis-(3-ethylbenzothiazoline-6-sulfonic acid) (ABTS^+^) is generated by the oxidation of ABTS with potassium persulfate before the addition of the antioxidant [48,49]. The decolorization of the blue/green (ABTS^+^) chromophore was measured as the percentage inhibition of absorbance at 734 nm and calculated in relation to the reactivity of Trolox (6-hydroxy-2,5,7,8-tetramethylchroman-2-carboxylic acid) as the standard under the same conditions. The (ABTS^+^) solution was diluted with phosphate buffered saline, pH 7.4, to an absorbance of 0.70 (±0.02) at 734 nm and equilibrated at 30 °C. After the addition of 1.0 mL of diluted (ABTS^+^) solution to 10 mL of sample, the absorbance reading was taken exactly 1 min after the initial mixing and up to 6 min. Samples were analyzed at three different dilutions within the linearity range of the assay. Results were expressed as Trolox equivalents antioxidant capacity (TEAC). TEAC is the concentration of Trolox required to give the same antioxidant capacity as the 1 mM test substance.

### 3.7. Statistical Analysis

Data were expressed as the mean ± standard deviation (SD) of the mean. The differences between extracts were evaluated by the one-way analysis of variance (ANOVA) test completed with a multi-comparison Tukey’s test.

## 4. Conclusions

A concentrated juice with high nutritional and organoleptic profile was produced from a blend of red fruit juices through an integrated membrane system based on a sequential combination of microfiltration, nanofiltration, and osmotic distillation. The experimental results clearly confirm that the content of bioactive compounds as well as the antioxidant activity of the original juice is very well-preserved in the concentrated juice independently of the degree of concentration achieved. In addition, the OD retentate at 60 °Brix resulted in the most active sample against pancreatic lipase and α-amylase inhibitory activity with IC_50_ values of 44.36 ± 0.85 and 214.65 ± 2.03 μg/mL, respectively.

The preservation of quality parameters in the concentrated juice clearly supported an excellent resistance of the OD membrane to wetting phenomena.

The appearance of concentrated juice was similar to that of fruit syrups, sugary products normally stored and marketed at room temperature. It can be added to water and pasteurized MF retentate (MF membranes retain microorganisms that must be inactivated) to obtain a juice with sensory, organoleptic, and nutritional properties similar to those of fresh juice. Alternatively, the concentrated product can be used as a flavoring and/or coloring for food products: it satisfies the functional characteristics of natural dyes such as high dyeing efficiency, good stability, and attractive color. The development of formulations for nutraceutical applications (anti-cholesterol, antiviral, antimicrobial, anti-inflammatory, antihypertensive products) is also of great interest.

## Figures and Tables

**Table 1:** Analyses of the total soluble solids (TSS), suspended solids (SS), total color density (TCD), pH, and conductivity in the samples of red fruit juice processed by MF, NF, and OD.

Sample	TSS (°Brix)	TCD	SS (mg GAE/L)	pH (ppm)	Conductivity (mS/cm)
Feed MF	13.8 ^a^	1.92 ± 0.01 ^a^	7.93 ± 0.15 ^ns^	3.59 ± 0.07 ^a^	3.89 ± 0.07 ^a^
Permeate MF (feed NF)	13.0 ^a^	1.99 ± 0.03 ^a^	n.d.	3.65 ± 0.07 ^b^	4.18 ± 0.08 ^b^
Retentate NF (feed OD)	33.0 ^b^	2.06 ± 0.03 ^^b^	n.d.	3.65 ± 0.07 ^^b^	3.77 ± 0.07 ^^a^
Retentate OD (1)	50.0 ^c^	2.23 ± 0.05 ^^b^	n.d.	3.65 ± 0.07 ^^b^	3.70 ± 0.07 ^^a^
Retentate OD (2)	60.0 ^d^	2.55 ± 0.01 ^^b^	n.d.	3.65 ± 0.07 ^^b^	3.78 ± 0.07 ^^a^
Sign.	**	**	ns	**	**

^ Samples diluted at 13 °Brix. Sign: significant; ^ns^: not significant. Differences between extracts were evaluated by one-way analysis of variance (ANOVA) test completed with a multi-comparison Tukey’s test. ** *p* < 0.01. Means in the same column with different small letters differ significantly.

**Table 2:** Analyses of betalains in the samples of red fruit juice processed by MF, NF, and OD (Bc, betacyanins; Bx, betaxhantins).

Sample	Bc (mg/L)	Bx (mg/L)	Bc (%)	Bx (%)
Feed MF	7.54 ± 0.07 ^b^	6.67 ± 0.09 ^a^	53.1 ^e^	46.9 ^a^
Permeate MF (feed NF)	6.85 ± 0.04 ^a^	6.75 ± 0.14 ^a^	50.3 ^d^	49.7 ^b^
Retentate NF (feed OD) ^	6.67 ± 0.25 ^a^	8.62 ± 0.21 ^c^	43.6 ^a^	56.4 ^e^
Retentate OD (1) ^	6.58 ± 0.16 ^a^	7.89 ± 0.17 ^b^	45.5 ^b^	54.5 ^d^
Retentate OD (2) ^	7.40 ± 0.09 ^b^	8.55 ± 0.13 ^c^	46.4 ^c^	53.6 ^c^
Sign.	**	**	**	**

^ Samples diluted at 13 °Brix. Sign: significant. Differences between extracts were evaluated by one-way analysis of variance (ANOVA) test completed with a multi-comparison Tukey’s test. ** *p* < 0.01. Means in the same column with different small letters differ significantly.

**Table 3:** Analyses of the polyphenols, flavonoids, and anthocyanins in the samples of red fruit juice processed by MF, NF, and OD.

Sample	Polyphenols (mg GAE/L)	Flavonoids (ppm)	Anthocyanins (ppm)
Feed MF	1370.20 ± 27.27 ^e^	422.31 ± 11.80 ^d^	407.99 ± 66.74 ^d^
Permeate MF (feed NF)	1341.96 ± 46.72 ^c^	413.33 ± 13.81 ^c^	362.51 ± 15.37 ^c^
Retentate NF (feed OD) ^	1331.76 ± 53.18 ^b^	397.10 ± 15.27 ^a^	318.72 ± 25.70 ^b^
Retentate OD (1) ^	1321.56 ± 55.60 ^a^	396.81 ± 6.75 ^a^	314.65 ± 26.99 ^a^
Retentate OD (2) ^	1363.92 ± 36.47 ^d^	400.00 ± 9.80 ^b^	312.61 ± 24.46 ^a^
Sign.	**	**	**

^ Samples diluted at 13 °Brix. Sign: significant. Differences between extracts were evaluated by one-way analysis of variance (ANOVA) test completed with a multi-comparison Tukey’s test. ** *p* < 0.01. Means in the same column with different small letters differ significantly.

**Table 4:** Inhibitory activity (IC_50_ μg/mL) of red juices against α-amylase and lipase.

Sample	Lipase	α-Amylase
Feed MF	48.82 ± 1.28 ^c^	304.06 ± 3.61 ^f^
Permeate MF (feed NF)	46.35 ± 1.16 ^b^	288.31 ± 2.76 ^a^
Retentate NF (feed OD)	49.41 ± 0.67 ^d^	258.16 ± 1.74 ^d^
Retentate OD (1)	44.80 ± 1.23 ^a^	252.62 ± 2.25 ^c^
Retentate OD (2)	44.36 ± 0.85 ^a^	214.65 ± 2.03 ^b^
Sign.	**	**

Data are expressed as means ± S.D. (*n*= 3). Acarbose was used as the positive control in the α-amylase test (IC50 value of 50.18 ± 1.32 μg/mL). Orlistat was used as positive control in lipase test (IC_50_ value of 37.15 ± 1.17 μg/mL). Sign: significant. Differences between extracts were evaluated by one-way analysis of variance (ANOVA) test completed with a multi-comparison Tukey’s test. ** *p* < 0.01. Means in the same column with different small letters differ significantly.

## Data Availability

Data are contained within the article.
